# Rapidly Processed Stool Swabs Approximate Stool Microbiota Profiles

**DOI:** 10.1128/mSphere.00208-19

**Published:** 2019-04-10

**Authors:** Nicholas A. Bokulich, Juan Maldonado, Dae-Wook Kang, Rosa Krajmalnik-Brown, J. Gregory Caporaso

**Affiliations:** a Center for Applied Microbiome Science, Pathogen and Microbiome Institute, Northern Arizona University, Flagstaff, Arizona, USA; b Biodesign Swette Center for Environmental Biotechnology, Arizona State University, Tempe, Arizona, USA; c Biodesign Center for Fundamental and Applied Microbiomics, Arizona State University, Tempe, Arizona, USA; d ASU Genomics Core, Biodesign Institute, Arizona State University, Tempe, Arizona, USA; e School of Sustainable Engineering and the Built Environment, Arizona State University, Tempe, Arizona, USA; f Department of Biological Sciences, Northern Arizona University, Flagstaff, Arizona, USA; University of Wisconsin—Madison

**Keywords:** 16S rRNA genes, gut microbiome, sample collection

## Abstract

Studies of the intestinal microbiome commonly utilize stool samples to measure the microbial composition in the distal gut. However, collection of stool can be difficult from some subjects under certain experimental conditions. Sampling of fecal material using sterile swabs can streamline sample collection, handling, and processing. In this study, we validate the use of swabs of fecal matter to approximate measurements of microbiota in stool using 16S rRNA gene Illumina amplicon sequencing and evaluate the effects of shipping time at ambient temperatures on accuracy. The results indicate that swab samples reliably replicate the stool microbiota bacterial composition, alpha diversity, and beta diversity when swabs are processed quickly (≤2 days) but that sample quality quickly degrades after this period and is accompanied by increased abundances of Enterobacteriaceae. Fresh swabs appear to be a viable alternative to stool sampling when standard collection methods are challenging, but extended exposure to ambient temperatures prior to processing threatens sample integrity.

**IMPORTANCE** Collection of fecal swab samples simplifies handling, processing, and archiving compared to collection of stool. This study confirms that fecal swabs reliably replicate the bacterial composition and diversity of stool samples, provided that the swabs are processed shortly after collection. These findings support the use of fecal swabs, when shipping and handling are done properly, to streamline measurements of intestinal microbiota.

## Abstract (precis)

Collection of fecal swab samples simplifies handling, processing, and archiving compared to collection of stool. This study confirms that fecal swabs reliably replicate the bacterial composition and diversity of stool samples, provided that the swabs are processed shortly after collection. These findings support the use of fecal swabs, when shipping and handling are done properly, to streamline measurements of intestinal microbiota.

## INTRODUCTION

The microbial communities inhabiting the human gastrointestinal tract play important roles in digestion, immune and metabolic regulation, and disease (1). Monitoring the gut microbiota is often performed to assess the impact of disease or other disturbances (2), therapeutic interventions (3), or host development (4). Measurements of the microbiota composition in the distal gut commonly utilize stool samples.

Collection and transport of stool may be difficult or impossible, however, under certain conditions, e.g., due to stool consistency or if subjects are unable or unwilling to provide stool. In a study by Sinha et al., the microbial compositions of stool swabs moderately correlated with those of stool (5); however, this study assessed the similarity of swab microbiota to stool at only three different storage times (frozen immediately or frozen after 1 day or after 4 days at room temperature). With a similar approach, Bassis and coworkers showed that the microbiota composition determined after collecting and immediately processing rectal swabs also approximated the stool microbiota composition (6). Rectal swabs are collected by insertion of a sterile swab into the rectum; fecal swabs are collected by applying a sterile swab to freshly passed stool or toilet paper. Collection of fecal swabs represents a simpler and less disruptive approach than either stool collection or rectal swabbing, permitting its use with sensitive patients. Swab collection also simplifies sample handling and processing during collection, archiving, and DNA extraction. This facilitates sampling under busy clinical settings or by individual subjects at home.

To validate stool swabs for measurements of intestinal microbiota, stool swabs and stool samples were collected from subjects in a previously published microbiota transfer therapy study (treatment that included a fecal transplant) (3). Both swabs and stool samples were collected from the same stool, and the microbiota composition and diversity were compared between sample pairs using 16S rRNA gene amplicon sequencing and analysis in the QIIME 2 software package (7). We show that swab and stool samples exhibit highly similar microbiota profiles, provided that the swabs are received and processed within 2 days of collection.

## RESULTS

**FIG 1:**
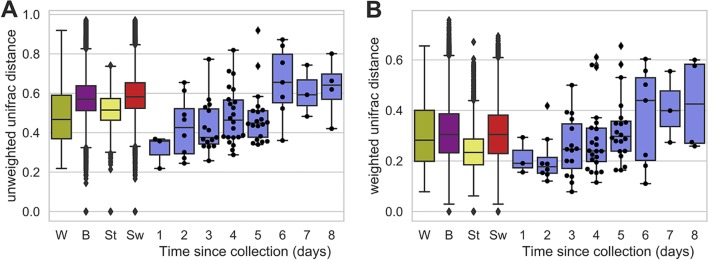
Unweighted (A) and weighted (B) UniFrac distance distributions between sample pairs. Box plots compare the pairwise distance distributions between all samples collected from within each individual subject (W; green; *n* = 98 pairwise distances), between all subjects (B; purple; *n* = 90,853), between all stool samples (St; yellow; *n* = 5,565), or between all swab samples (Sw; red; *n* = 51,681) collected from the same subject at different times and between pairs of stool and swab samples collected from the same individual at the same time (paired samples, shown in blue; sample sizes are shown by shipping time in Table 1). Swarm plots are overlaid for paired distance measurements between swab and stool samples only, indicating the actual distribution of paired distances.

In the original study reanalyzed here, samples were collected from children with autism spectrum disorders (ASD) (*n* = 18) and neurotypical children (*n* = 20) (mean age, 11.1 ± 2.7 years) across an 18-week period (10 weeks of the clinical trial and an 8-week follow-up) (3). Stool samples were collected at 4 time points (baseline, week 3, week 10, and week 18) and frozen immediately after collection. Swabs were collected at 11 time points (every 2 weeks since baseline and week 3), including the time points when stool was collected, and shipped to the laboratory by standard postal mail. Information on the study population and samples is shown in Table 1. Our current study focused on swab samples and stool samples collected from the same individual subject at the same time (*n* = 82), but additional stool and swab samples were included for measuring pairwise distances between all stool and swab samples collected from the study population, as described below (Fig. 1).

**TABLE 1:** Overview of study population

Characteristic	Value
No. of patients in the following group:	
Neurotypical	20
ASD	18
No. of patients by sex	
Male	34
Female	4
Mean ± SD age (yr)	11.1 ± 2.7
No. of samples of the following type:	
Stool	105
Swab	321
Stool + swab pairs	82
No. of samples with the following shipping time (days)^*a*^:	
1	3
2	8
3	15
4	22
5	20
6	7
7	3
8	4
Mean ± SD avg temp (°C) when samples were^*b*^:	
Sent	15.0 ± 3.6
Received	14.9 ± 3.6
No. of samples with the following mo of collection:	
January	123
February	70
March	88
April	67
May	12
June	2
July	1
August	0
September	0
October	30
November	12
December	115

a Shipping time is shown only for swab samples that were paired to a stool sample in this study (*n* = 82).

b Average daily outdoor temperature at the time of collection and receipt for swabs only. Data were collected from the U.S. National Weather Service (https://www.weather.gov/).

An accurate measurement of intestinal microbiota composition should demonstrate a high degree of similarity to stool composition, the current gold standard method. We measured phylogenetic similarity between samples using abundance-weighted and unweighted pairwise UniFrac distances (8). We also measured paired differences in the observed richness of sequence variants, phylogenetic diversity (PD) (9), and Shannon diversity and evenness to assess alpha diversity differences between swab and stool samples.

### Fresh swab microbiota resembles stool microbiota.

Freshly processed (≤2 days) pairs of stool and swab samples collected from the same individual at the same time (paired samples; *n* = 11) were significantly more similar to each other than stool or swab samples collected from the same individual but at different times (within-subject pairs; *n* = 98), suggesting that stool and swab samples yield similar community structures when swabs are processed quickly (Fig. 1) (Mann-Whitney U test, weighted UniFrac, *U* = 294.5 and *P* = 0.007; unweighted UniFrac, *U* = 342.5 and *P* = 0.024). Swabs experiencing longer transport times (*n* = 71) were not significantly more similar to their stool pairs than they were to within-subject pairs (*P* > 0.05), suggesting that the microbiome after shipping times of longer than 2 days does not reliably represent the microbiome of stool samples frozen at the time of collection.

### Transport time degrades swab accuracy.

Both unweighted and weighted UniFrac paired-sample distances increased as swab shipping time increased (Fig. 1), becoming significantly more dissimilar than within-subject pairs by 6 days of shipping (Wilcoxon signed-rank test, *P* < 0.05); transport time was positively correlated with paired-sample dissimilarity for both weighted UniFrac (Spearman correlation coefficient [ρ] = 0.88, *P* = 0.004) and unweighted UniFrac (Spearman ρ = 0.88, *P* = 0.004) distances. Thus, transport times longer than 1 to 2 days appear to have a damaging effect on swab compositional accuracy, similar to the negative effects of room temperature storage on the compositional accuracy of stool samples not stored in preservative (10).

**FIG 2:**
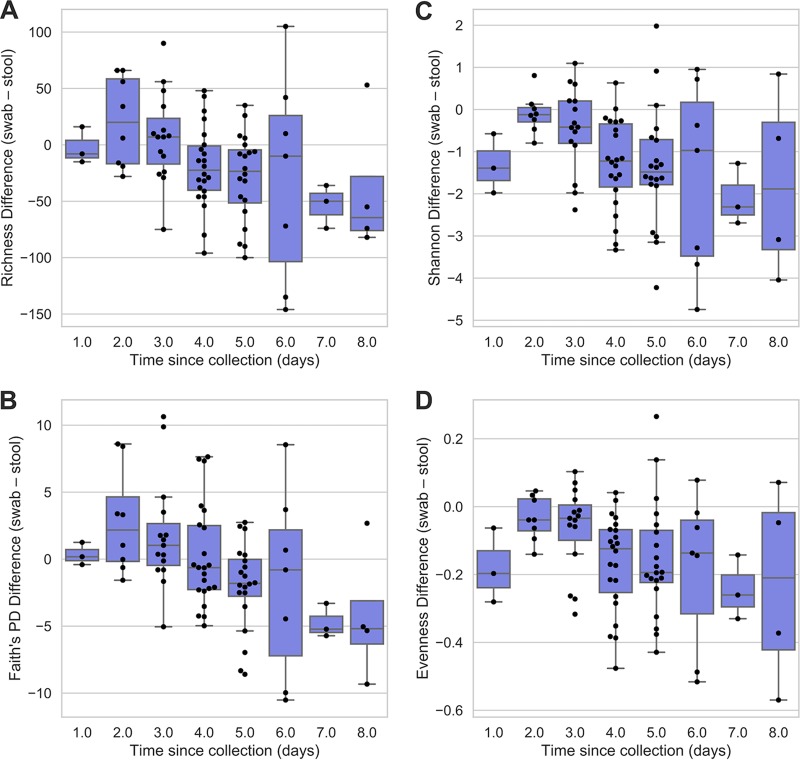
Observed differences in alpha diversity metrics between stool and swab paired samples in relation to transport time. Box plots show quartile distributions of differences between paired samples (swab − stool observed diversity) for observed richness (A), Shannon H (B), Faith’s PD (C), and evenness (D). Swarm plots are overlaid to show the actual distribution of metric differences. Sample sizes are shown by shipping time in Table 1.

Pairwise differences in alpha diversity between paired samples (swab-stool observed diversity; *n* = 82) indicated that swab microbiota richness (Spearman ρ = −0.86, *P* = 0.006) and PD (Spearman ρ = −0.88, *P* = 0.004) decreased as the transport time increased. Shannon diversity (Spearman ρ = −0.64, *P* = 0.086) and evenness (Spearman ρ = −0.57, *P* = 0.139) also decreased with increasing transport time, but the correlations were not significant (Fig. 2). After 4 days of transport time, swab richness, Shannon diversity, and evenness, but not PD, were significantly lower than those for stool (Wilcoxon signed-rank test, *P* < 0.05), but transport times under 4 days did not significantly impact these alpha diversity metrics (*P* > 0.05). Swabs experiencing longer transport times (6 to 8 days) were not significantly different from their stool pairs, though this appears to be due to the small sample sizes and very high variances observed with these transport times. The time-dependent decrease in richness and evenness likely indicates that the growth of one or more bacterial species (facultatively aerobic enterobacteria, as the results presented below suggest) numerically overshadows the abundance of other bacteria (e.g., strict anaerobes and slower-growing organisms). The latter organisms do not disappear from this closed system but become less likely to be detected.

### Supervised learning classification confirms accuracy of fresh swabs.

To confirm the similarity of swab microbiota compared to stool microbiota, we used random forest (11) classification models to predict the sample type (stool or swab) based on the microbiota composition (16S rRNA gene sequence variants). Stool samples were compared to swab samples exposed to between 3 and 8 days of transport time (where swab samples were highly dissimilar to stool) or only 1 to 2 days of transport time (where swab samples were more similar to stool). Swabs exposed to 3 to 8 days of transport time could accurately be classified 94.6% of the time and stool samples could accurately be classified 90.1% of the time. However, swabs exposed to ≤2 days of transport time could not be reliably distinguished from stool samples: swab samples were correctly classified only 47.1% of the time (random chance is 50%). Notably, the most important features identified in each model were members of the family Enterobacteriaceae.

### Swabs are characterized by overrepresentation of Enterobacteriaceae compared to stool samples.

**FIG 3:**
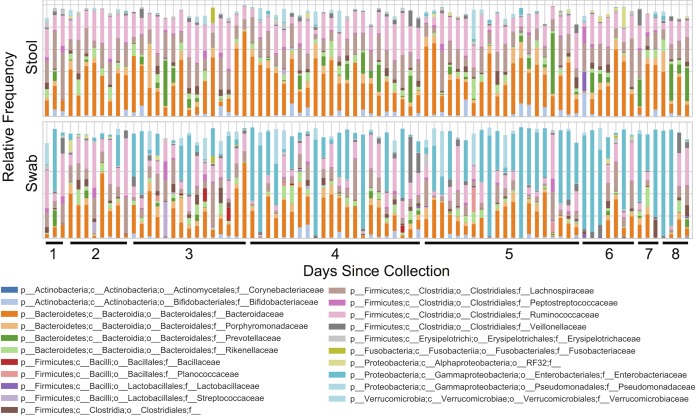
Relative abundance of bacterial families in paired stool (top) and swab samples (bottom). Paired stool and swab samples collected from the same individual at the same time point are aligned along the *x* axis and sorted by swab transport time. p, phylum; c, class; o, order; f, family.

**FIG 4:**
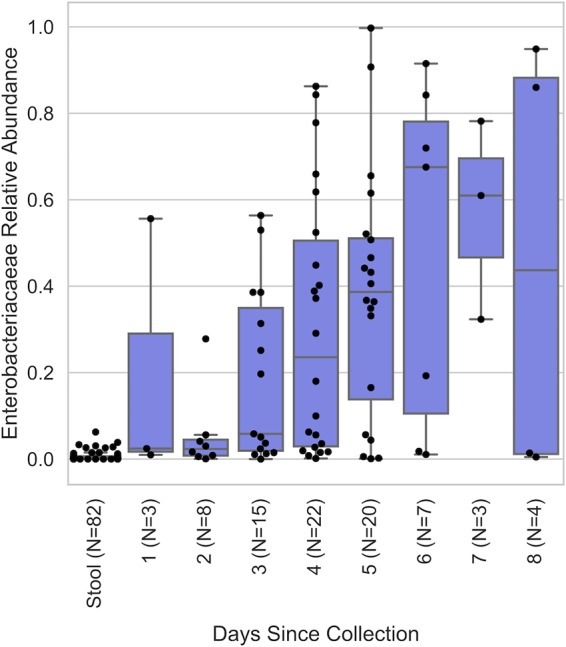
Distribution of Enterobacteriaceae relative frequencies in stool samples and in swab samples exposed to different transport times.

Next, we determined the impact of transport time on the bacterial taxonomic composition in swabs compared to that in stool to identify the taxa responsible for altered diversity patterns. The taxonomic compositions of swab samples became dominated by Enterobacteriaceae as the transport time increased, leading to a notable disparity compared to that for stool samples collected from the same subject at the same time (Fig. 3). Enterobacteriaceae relative abundance was positively correlated with transport time (Spearman ρ = 0.88 [*P* = 0.004]; Pearson *R* = 0.87 [*P* = 0.005]) (Fig. 4). Notably, two samples received after 1 to 2 days of transport also had relatively higher abundances of Enterobacteriaceae (Fig. 4).

**FIG 5:**
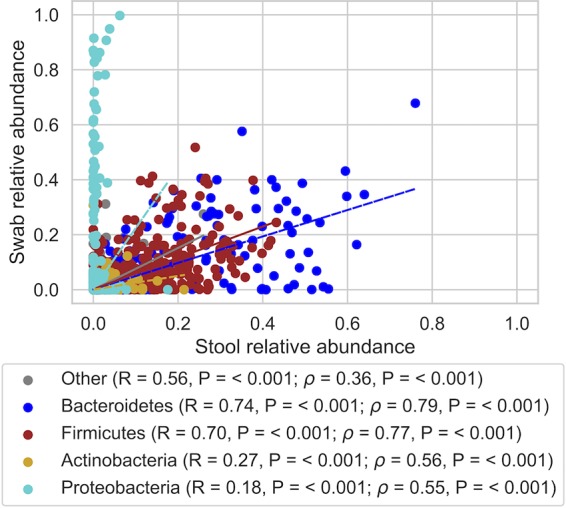
Scatter plot comparing the relative abundances of all taxa observed in stool and swab samples. Taxa are colored by their phylum affiliation (all other phyla are combined into “other”), and linear regressions for each phylum are plotted. Pearson *R* and Spearman (ρ) correlation coefficients and their *P* values comparing stool and swab abundances for each phylum are shown in the key.

Paired analysis of composition of microbiomes (ANCOM) tests (12) between all paired samples (regardless of transport time) indicated that bacterial species in the families Enterobacteriaceae and *Bacillaceae* were overrepresented in swab samples (*P < *0.05) and a broad range of *Clostridiales* were overrepresented in stool (Table 2). While the phylum *Proteobacteria* (represented mostly by the family Enterobacteriaceae) was overrepresented in swab samples compared to their matching stool samples (slope > 1), most other phyla exhibited slight overrepresentation in stool (slope < 1) (Fig. 5). Nevertheless, the abundances of all phyla were significantly correlated between swabs and their matching stool samples (Spearman ρ = 0.67, *P* < 0.001) (Fig. 5). This most likely indicates the cellular growth of Enterobacteriaceae while other populations remain largely static and are supplanted at an approximately even rate. This could also indicate death and DNA degradation of these other populations, but that scenario seems much less likely, given the short time frame of this experiment; however, we cannot discern changes to absolute abundance based on our compositional (relative abundance) sequence data.

**TABLE 2:** ANCOM differentially abundant sequence variants^*d*^ between stool and swab paired samples

Feature identifier^*a*^	Family	Genus or genus and species^*b*^	*W*^*c*^	Sequence abundance			
				Stool median	Stool maximum	Swab median	Swab maximum
f3fc3c1992d8118d6105048408aaf6d6	Enterobacteriaceae		2,457	27.5	1,932	2,201.5	57,802
8ce638638fc5ee9e2128ac4bd03ed11e	Enterobacteriaceae	*Klebsiella*	2,455	1	10	1	35,068
5a83ea3d76cd341dac86f333c7d5f293	Enterobacteriaceae	*Citrobacter*	2,436	1	18	1	18,276
c57bf51f33c656b83ae967392536b842	Enterobacteriaceae	*Klebsiella*	2,406	1	66	1	3,235
801cc2f4b3dfb4b130c4ba7ef4a20094	*Bacillaceae*	*Bacillus*	2,276	1	1	1	2,076
fb9c4b48fcb5d89827e4d868e63846a8	*Lachnospiraceae*	*Blautia*	2,213	169	4,721	73.5	2,374
2f561a0913fb0ed1a03d6cbdd1796e0c	*Lachnospiraceae*	*Coprococcus*	2,294	122.5	2,295	50.5	1,321
edfefd945764652423a9183e4934f63e	*Lachnospiraceae*	*Roseburia*	2,229	38	1,327	1	769
c4e55d1fa1d9152699f44847eec89821	*Lachnospiraceae*		2,375	152.5	1,544	46.5	701
6f063a38df307a2c50a525bf2ae85f7d	*Lachnospiraceae*	*Blautia*	2,273	78	1,996	34	536
8be4f08a4c290c121885c6d3abc32186	*Ruminococcaceae*	*Oscillospira*	2,215	13.5	1,217	1	455
b54e516c620e7b11f1f267f154efe1f6	*Lachnospiraceae*		2,212	13	464	1	150
4949d5468cabaae7de1a985e6a479a6a	*Lachnospiraceae*	Coprococcus catus	2,234	14.5	154	1	146
ebf3c3237392738d0fdeb35e9bb35bcd	*Alcaligenaceae*	*Sutterella*	2,407	21	1,527	1	137
efeef69c255be9b873b917707495b22f	*Lachnospiraceae*		2,243	1	154	1	105
2d1be5a482c6d0a6b58a5d9b5f3c5b3d	*Ruminococcaceae*	*Oscillospira*	2,355	24	235	1	101
40a904445b77cf5125c51fb01f785193	*Lachnospiraceae*		2,248	1	279	1	97

a Feature identities equal the MD5 hashes of the 16S rRNA gene sequences identified as being differentially abundant between paired stool and swab samples.

b Genus and species names are shown where available. Any feature missing a genus and/or a species label was classified as belonging to a species that is missing a genus and/or a species annotation in the Greengenes 16S rRNA gene sequence reference database.

c *W* equals the number of ANCOM subhypotheses that have passed for each individual taxon, indicating that the ratios of that taxon’s relative abundance to the relative abundances of *W* other taxa were detected to be significantly different between stool and swab samples.

d Sequence variants differentially abundant at a *P* value of <0.05.

## DISCUSSION

This study has demonstrated the accuracy of swabs for approximating the composition of stool samples and evaluated the effect of transport time. Previous authors have examined the reproducibility and accuracy of fresh swabs for approximating stool microbiota measurements (5). We extend these prior studies by demonstrating the impact of storage time on swab similarity to stool. This corroborates earlier findings that swab and stool samples yield similar biological conclusions (3, 5).

We show that swabs provide an accurate approximation of stool microbiota diversity, composition, and structure, provided that the swabs are processed when they are as fresh as possible (≤2 days). Stool samples and swabs could not be reliably distinguished by supervised learning classification, indicating the close resemblance between these collection methods. Long transport times were associated with the overrepresentation of Enterobacteriaceae (probably due to growth under aerobic conditions), decreasing the accuracy of the microbiota profiles. Prospectively, this finding could be used to further enhance the use of swabs for fecal microbiota profiling. Except in scenarios where high levels of Enterobacteriaceae are a normal constituent of the intestinal microbiota, such as following gastric bypass surgery (13, 14), Enterobacteriaceae could be used as a marker for validating swab integrity, e.g., to reject outliers that may have experienced inadequate shipping or storage. For example, in our study, 2 out of 11 swab samples received within 2 days of collection still exhibited outlying relative abundances of Enterobacteriaceae (Fig. 4), indicating that transport time alone cannot ensure sample integrity. Modeling of the compositional changes over time could support the development of algorithms to correct for biases arising from collection and storage issues, e.g., to adjust Enterobacteriaceae relative abundance based on storage/transport time and temperature.

Blooms of *Gammaproteobacteria* were reported in a previous meta-analysis by Amir et al. (15) that compared the microbial community composition of swabs that were shipped at uncontrolled temperatures versus swabs that were frozen immediately. However, that study did not examine the relationship between swab shipping time and *Gammaproteobacteria* abundance or other microbiome characteristics, as was measured in our current study, though their meta-analysis did include published studies examining the impact of room temperature storage on stool microbiota. Amir et al. (15) recommended filtering specific gammaproteobacterial sequence variants to rectify microbiome measurements from swabs stored at room temperature. However, this approach is highly specific to their study methodology; e.g., those blooming sequence variants may not generalize to different read lengths, to sequencing technologies, or to studies not included in their meta-analysis. Hence, further studies are needed to devise general strategies for mitigating room temperature storage-related blooms.

Our study is limited by the relatively small number of subjects (*n* = 38) (Table 1). The study cohort included only children, several of whom had received a microbiota transfer therapy. Swabs collected from subjects with highly distinct stool microbiota (e.g., subjects suffering from severe gastrointestinal illness) could exhibit storage profiles different from those observed in our study and should be revalidated for atypical study cohorts. This study only examined swabs that were shipped by mail. Hence, results may be impacted by temperature fluctuations that were not controlled for in this experiment. Additionally, multiple shipping times were not tested for each individual swab sample; collection and exposure of replicates to different shipping times would enable repeated measurements to assess longitudinal changes in individual taxa. Given the unpredictability of shipping times by standard post, these results underscore the importance of utilizing expedited post, temperature control, and/or preservation techniques for fecal swab collection if swabs cannot be frozen immediately upon collection.

Stool collection is not always easy or convenient. This may be due to logistical constraints (e.g., at-home collection or busy clinical settings), sample characteristics (e.g., fecal incontinence), or subject comfort. Stool swabs represent a viable alternative for measurement of distal gut microbial composition and diversity. Swabs are also considerably easier to handle and process than stool samples, streamlining collection and DNA extraction protocols. Although we found that stool and fresh swab samples could not be reliably distinguished by supervised learning classification, we do not recommend mixing stool and swab collection methods within the same study, in order to avoid introduction of experimental variation and potential sampling biases. For example, contamination and other artifactual biases could differ between collection methods and different brands of swabs, and variation should be minimized as much as possible. In studies where different collection methods become necessary, investigators should test to ensure that collection methods do not covary with other sample characteristics or metadata.

## MATERIALS AND METHODS

### Sample collection and processing.

Stool samples and swabs were collected and processed as previously described in a study of children with ASD receiving microbiota transfer therapy (3). Stool samples and fecal swabs were collected by the subjects’ parents. Stool samples were stored in dry ice, collected by a driver, and frozen at −80°C immediately upon arrival at the laboratory. Swabs were shipped to the lab by standard postal mail. After defecation, fecal matter was collected from toilet paper using a sterile swab (Fisher Scientific BD culture swab item number B4320135), taking care not to contact the paper or overload the swab. Samples were shipped at room temperature and frozen at −80°C immediately upon arrival at the laboratory. Swab samples were primarily shipped within Arizona at different times of year, so temperatures (and, hence, shipping effects) may be slightly higher than those in other regions. The time between shipping and receipt was logged as the number of days in transit, which was used to perform the statistical analyses described below. DNA extraction and sequencing were performed as previously described, following the Earth Microbiome Project standard protocol for 16S V4 rRNA gene sequencing with 515f-806r primers (16). A total of 105 stool samples and 321 swab samples were collected and analyzed in the current study, including 82 pairs of stool and swab samples that were collected from the same source stool (Table 1). Swab transport times in the original study varied from 0 to 68 days; however, only days 1 to 8 contained a sufficient sample size (minimum, *n* = 3 stool-swab pairs), and these samples were used in the current study for assessing the impact of transport time on swab composition accuracy compared to that for paired stools.

### Microbiome analysis.

Sequence data were processed and analyzed using the QIIME 2 software package (7). Raw sequences were quality filtered using the DADA2 software package (17) to remove phiX, chimeric, and erroneous reads. Sequence variants were aligned using the mafft program (18) and used to construct a phylogenetic tree using the fasttree2 program (19). Taxonomy was assigned to sequence variants using q2-feature-classifier (20) against the Greengenes 16S rRNA reference database (13_8 release) (21).

### Statistical analysis.

QIIME 2 was used to measure the following microbiota alpha diversity metrics: richness (as observed sequence variants), Shannon diversity and evenness, and phylogenetic diversity (9). Microbiome beta diversity was estimated in QIIME 2 using weighted and unweighted UniFrac distances (8). Feature tables were evenly subsampled at 5,000 sequences per sample prior to alpha or beta diversity analyses.

Alpha diversity differences and UniFrac distances between paired stool and swab samples from identical source samples (paired samples) were calculated using paired Wilcoxon signed-rank and Mann-Whitney U tests as implemented in Scipy (https://scipy.org) and q2-longitudinal (22). ANCOM (12) was used to test whether the abundances of individual taxa differed between paired samples, as implemented in the QIIME 2 plug-in q2-composition (version 2017.4.0) with the following parameters: transform_function=clr (center log-ratio transformation); statistical_test=ttest_rel (paired-sample *t* test); the Holm-Bonferroni false discovery rate correction was applied by default. Spearman correlation coefficients were computed between transport time and median alpha diversity metrics, UniFrac distance, and Enterobacteriaceae relative abundance. Mann-Whitney U tests were used to test whether the relative abundances of the family Enterobacteriaceae were significantly different between stool samples and swab samples exposed to different transport times. Supervised learning classification was performed in q2-sample-classifier (23), using random forests classifiers (11) grown with 500 trees, trained on a random subset of the data (80%) and validated on the remaining samples.

### Data availability.

This study reanalyzed a previously published 16S rRNA gene sequence data set (3), which is available in the NCBI Sequence Read Archive under accession number PRJNA529598 and in the open-source microbiome database Qiita (qiita.microbio.me) under study identification number 10532.
